# Gut Microbiota and Targeted Biomarkers Analysis in Patients With Cognitive Impairment

**DOI:** 10.3389/fneur.2022.834403

**Published:** 2022-02-17

**Authors:** Shourong Lu, Ying Yang, Qiao Xu, Shuqiang Wang, Jie Yu, Bingshan Zhang, Zhuo Wang, Yunyun Zhang, Wenwei Lu, Kan Hong

**Affiliations:** ^1^Department of Geriatric, The Affiliated Wuxi People's Hospital of Nanjing Medical University, Wuxi, China; ^2^Department of Medicine, Wuxi Xin'an Community Health Service Center, Wuxi, China; ^3^Department of General Practice, The Affiliated Wuxi People's Hospital of Nanjing Medical University, Wuxi, China; ^4^State Key Laboratory of Food Science and Technology and School of Food Science and Technology, Jiangnan University, Wuxi, China

**Keywords:** cognitive impairment, gut microbiome, biomarkers, *Lactobacillus*, *Bifidobacterium*

## Abstract

Gut microbial alteration is closely associated with brain disorders including cognitive impairment (CI). Gut microbes have the potential to predicate the development of diseases. However, the gut microbial markers for CI remain to be elucidated. In this study, the gut microbial alterations were assessed using16S rRNA sequencing, and identified the gut microbial markers using a random forest model. The results showed that there were significant gut microbial differences between the control and CI groups based on beta diversity (*p* < 0.002). Patients with CI had higher abundances of Actinobacteria and Proteobacteria but lower proportions of Bcateroidetes and Firmicutes vs. that in the control group. Patients had 39 special genera and the control subjects had 11 special genera. Furthermore, 11 genera such as *Blautia, Roseburia*, and *Lactococcus* and 18 genera such as *Lactobacillus, Ruminococcus* 2, and *Akkermansia* were the differential taxa in the control and CI groups, respectively. Gene functions related to nutrient metabolisms were upregulated in patients with CI. This suggested that the huge differences in gut microbes between the two groups and gut microbiota had the potential to predicate the development of CI. Based on machine learning results, 15 genera such as *Lactobacillus, Bifidobacterium*, and *Akkermansia* were selected as the optimal marker set to predicate CI with an area under curve (AUC) value of 78.4%. The results revealed the gut microbial markers for CI and provided a potential diagnosis tool to prevent the development of CI in the elderly.

## Introduction

Cognitive impairment is closely associated with the development of psychiatric illnesses such as hypertension, depression, and Alzheimer's disease (1–3). Age is an important factor for cognitive impairment (CI) and there is increasing prevalence with the development of aging worldwide (4). Furthermore, educational degree, sleep disorder, and vitamin D intake are the risk factors for CI in elderly people (5–7). In the elderly, gastrointestinal tract and brain functions are gradually declining, and these factors are inevitable challenges for them. CI is the high-risk factor for dementia in elderly people, 10–30% of patients with mild CI and 20–66% of them convert into dementia within 1 and 2–4 years, respectively (8, 9). Dementia is difficult to reverse and there are no effective ways to treat it (10). CI leads to a huge burden for family and society. Therefore, the early prevention of CI is a key window to reduce the prevalence of dementia in the elderly.

Gut microbial alteration is closely associated with the development of aging in the elderly and affects their physiology functions including CI. The “Gut-brain” axis has been reported to solve the function barrier related to the brain and suggests bacteria play a key role in the development of neurodevelopmental disorders (11). Bacterial species such as *Limosilactobacillus reuteri, Bifidobacterium pseudolongum*, and *Lactobacillus johnsonii* are reduced in the maternal high-fat-diet offspring and negatively affect offspring social behavior (12). *Verrucomicrobiaceae* and unclassified Firmicutes are increased but *Prevotellaceae* and *Erysipelotrichaceae* are decreased, and they affect the β-glucuronate and tryptophan metabolism in the patients with Parkinson's disease (13). These results suggest that gut microbiota and their metabolism influence deeply the brain function and manipulate the host's emotion and behavioral intentions. Fecal microbiota transplant from aged donor mice into young recipients leads to impaired spatial learning and memory (14). This implies that gut microbiota is an important cause to induce CI symptoms.

In recent years, microbial marker severs as a non-invasive diagnosis tool for some diseases such as hepatocellular carcinoma, colorectal cancer, and type 2 diabetes (15–17). Gut microbial marker has the potential to predict the development of CI and may be an effective target tool to prevent CI in the elderly. In this study, a total of 60 fecal samples were collected from the patients with CI and control subjects. Combined 16S rRNA sequencing with machine learning revealed microbial biomarkers that predicted the development of CI and provided a potential diagnosis tool to prevent CI for the elderly.

## Materials and Methods

### Study Subjects

Patients (*n* = 33) with CI and controls (*n* = 27) aged more than 68 years were enrolled in the Xin'an community in Wuxi city. The study protocol was approved by the Ethics Committee of the Wuxi People's Hospital. Each subject provided written informed consent.

### CI Assessment

Mini-mental state examination (MMSE) index was used to assess the mental state and degree of CI for the subjects (27–30, normal cognitive function; <27, CI). The activities of daily living (ADL) index was used to evaluate the ability of daily living in family and the community in the elderly people (100, self-care; >60, basically self-care; 41–60, need assistance; 21–40, rely on others; <20, completely dependent on others).

### Baseline Clinical Characteristic Collection

Baseline clinical characteristics including gender, age, height, weight, education level, sleep duration, and occupation were collected during the clinical interview. Fecal samples were stored at −80°C until for use.

### 16S rRNA Amplicon Sequencing

Using the FastDNA Spin Kit (MP Biomedicals, Santa Ana, CA, USA), DNA was extracted for feces according to the instructions. The V3–V4 region was amplified with the primers (forward, 5′-CTCCTACGGGAGGCAGCA-3′; reverse, 5′-GGACTACHVGGGTWTCTAAT-3′). PCR products were purified by TIANgel Mini Purification Kit (TIANGEN, Beijing, China). After determination for DNA concentration and library construction, the amplified fragment was sequenced on an Illumina MiSeq PE300 platform (Illumina, Santiago, CA, USA). Sequencing results were analyzed using the QIIME2 pipeline. Non-metric multidimensional scaling (NMDS) was performed to evaluate change in beta diversity of gut microbiota based on the Bray-Curtis distance (18). Differential taxa were identified using the linear discriminant analysis effect size (LEfSe) (19). Phylogenetic investigation of communities by reconstruction of unobserved states (PICRUSt) was employed to predict the gene function of gut microbiota based on the Kyoto Encyclopedia of Genes and Genomes (KEGG) Orthology database between groups with and without CI (20). A random forest model was used to explore the microbial markers for CI (21). Sequence data were deposited in the Sequence Read Archive database as BioProject PRJNA789994.

### Statistical Analysis

The SPSS software version 20 (Chicago, Illinois, USA) and R software version 4.0.5 (open access, https://www.r-project.org/) were used to perform statistical analysis. The comparison was performed using an unpaired *t*-test between the control and CI groups. A *p* < 0.05 was indicated statistically significant.

## Results

### Baseline Characteristics of Subjects

**Figure 1:**
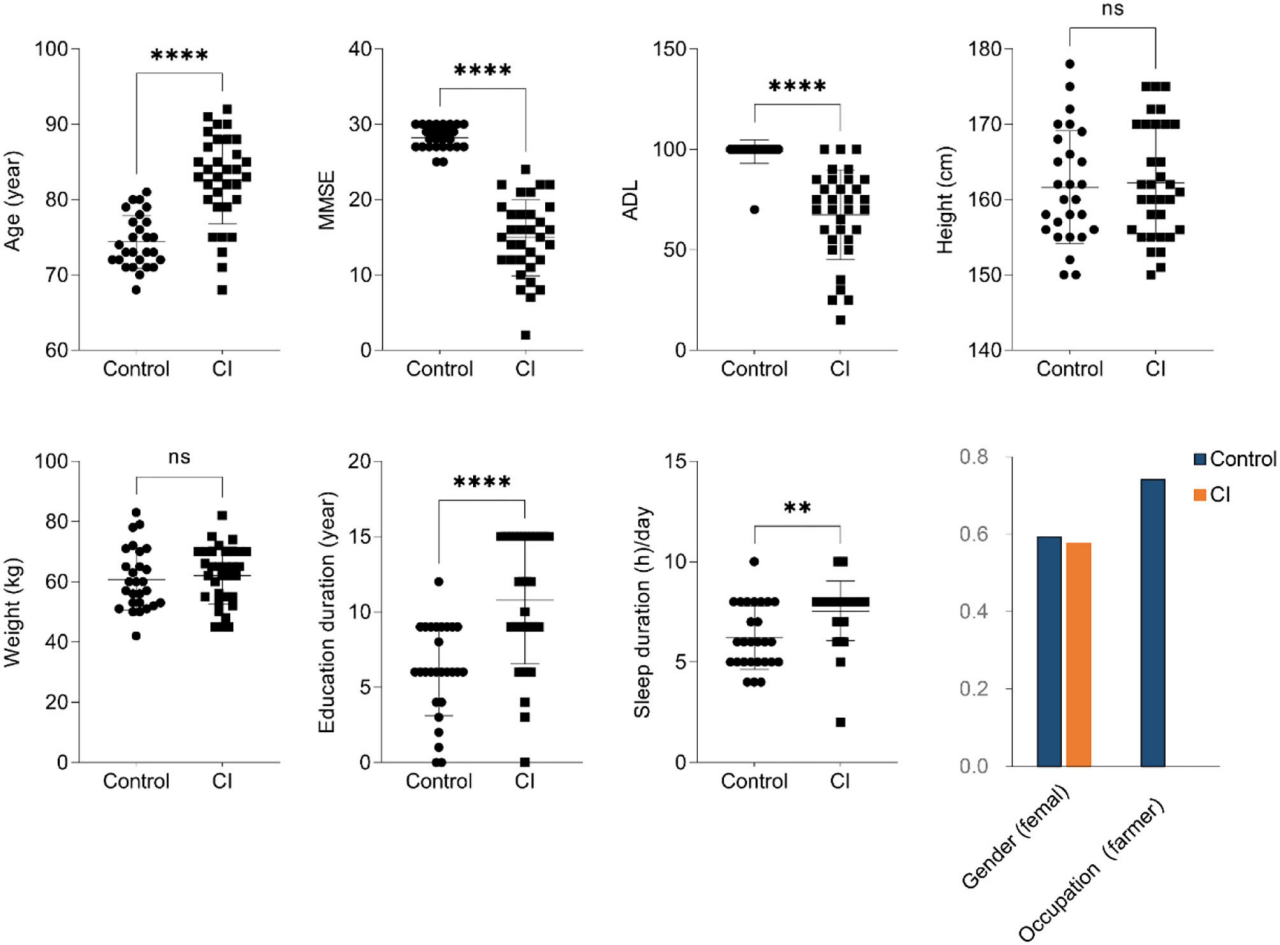
Baseline clinical characteristics (unpaired *t*-test, ns, no significance, ^**^p < 0.01, ^****^p < 0.0001).

In this trial, 33 patients with CI and 27 healthy controls were enrolled. As shown in Figure 1, there was no significant difference in gender, height, and weight between the two groups. Compared to the control group, patients with CI had lower MMSE and ADL indices but had higher age, education duration, and sleep duration per day in the CI group. Furthermore, patients in the CI group were all retirees but the farmer was the major occupation (74.07%) in the control group. The results showed that people with older age had a higher risk to suffer the CI symptoms although they had higher education duration and sleep duration in the CI group.

### Alteration of Gut Microbial Diversity

**Figure 2:**
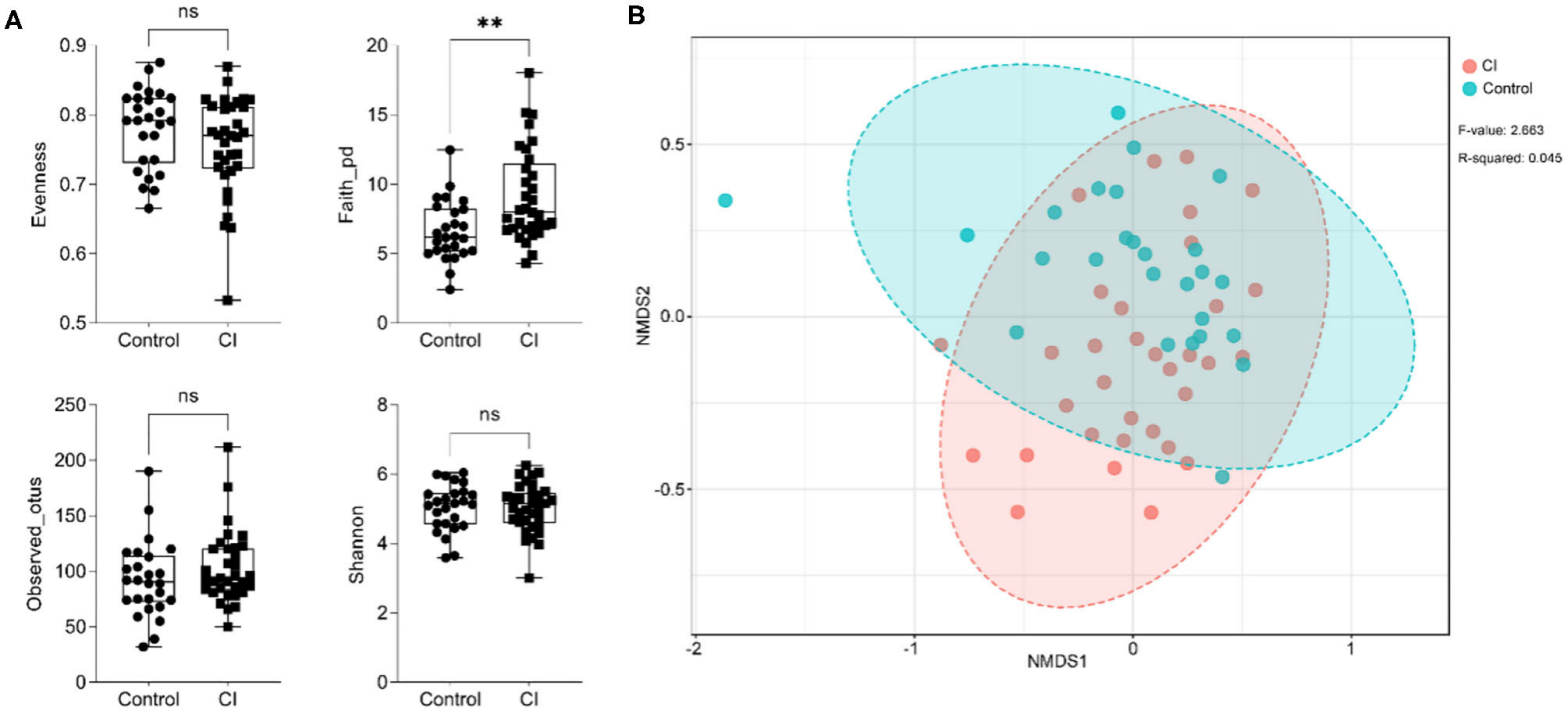
Changes in the alpha and beta diversities of gut microbes. **(A)** Alteration of alpha diversity (unpaired *t*-test, ns, no significance, ^**^p < 0.01). **(B)** Beta diversity alteration based on non-metric multidimensional scaling (NMDS) analysis.

To assess the alteration of gut microbial diversity including alpha and beta diversities in subjects, 16S ribosomal RNA sequencing was performed. For alpha diversity of gut microbes, there were no significant differences in evenness, observed_otus, and the Shannon indices except for the faith_pd index (Figure 2A). Compared to the control group, the faith_pd index was significantly higher. Furthermore, there was a difference in the beta diversity of gut microbiota between the two groups [Figure 2B, stress = 0.25, *p* < 0.002, permutational multivariate ANOVA (PERMANOVA)], and the development of CI was associated with alterations of gut microbial composition. The results showed that alpha diversity of gut microbiota was no significant alteration between the two groups but beta diversity representing gut microbial structure and composition had remarkable changes in the CI group vs. the control group.

### Changes in Gut Microbial Composition

**Figure 3:**
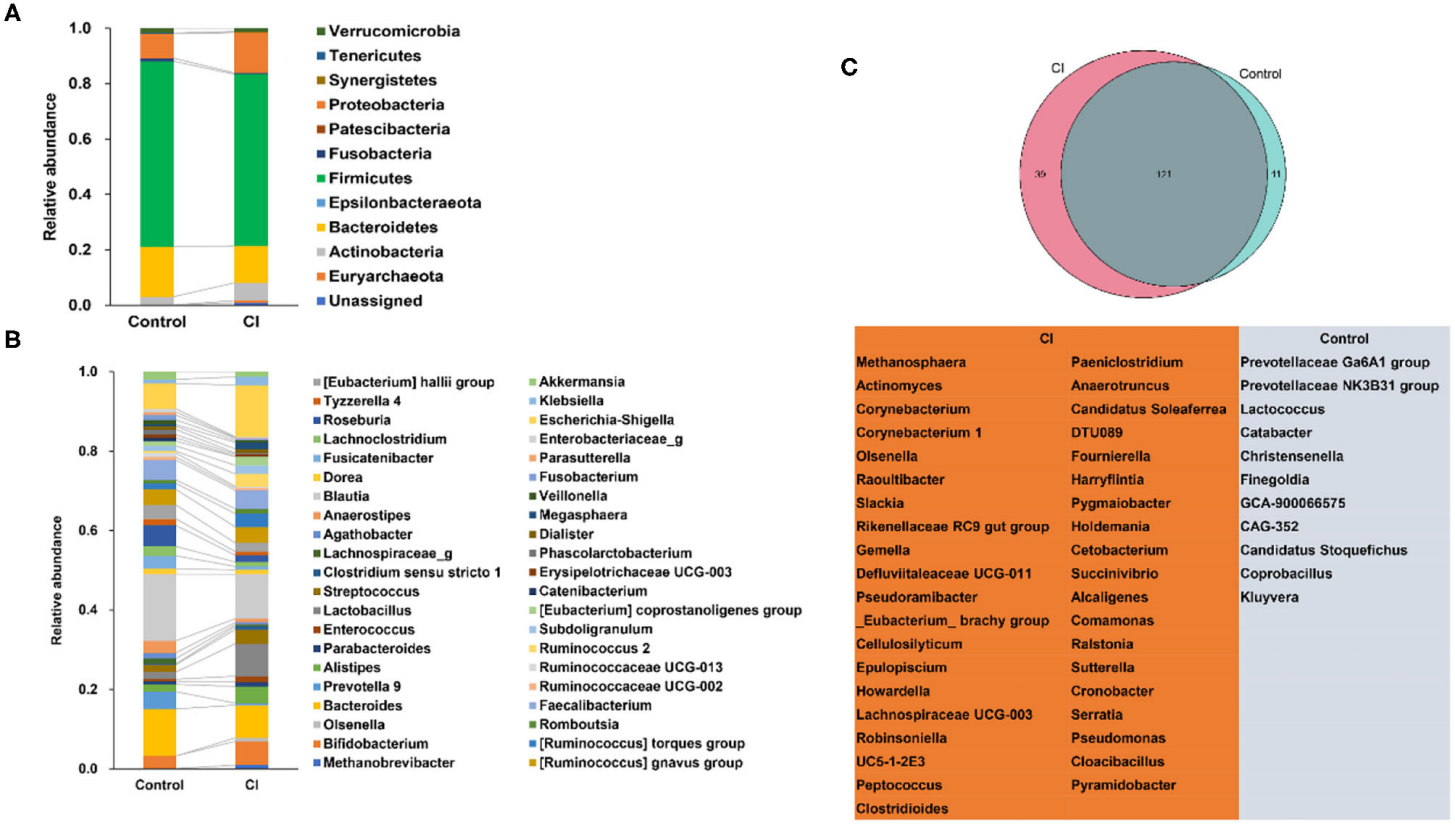
Alterations of gut microbial composition. **(A)** Changes in gut microbes at the phylum level. **(B)** Changes in gut microbes at the genus level. **(C)** The special genera in the control and cognitive impairment (CI) groups.

At the phylum level, patients had shown higher abundances of Actinobacteria and Proteobacteria but lower proportions of Bacteroidetes and Firmicutes in the CI group vs. that in the control group (Figure 3A). There were no significant differences in the abundances of Verrucomicrobia and Tenericutes between the two groups. Fusobacteria was enriched in the control group but Euryarchaeota was higher in the CI group. At the genus level (relative abundance >0.005 in any group), *Bifidobacterium, Alistipes, Lactobacillus, Streptococcus*, (*Ruminococcus*) *torques* group, *Ruminococcus* 2, *Escherichia-Shigella*, and *Klebsiella* were enriched in the CI group (Figure 3B). The relative abundances of *Bacteroides, Prevotella* 9, *Lachnospiraceae*_g, *Anaerostipes, Fusicatenibacter, Lachnoclostridium, Roseburia, Tyzzerella* 4, (*Eubacterium*) *hallii* group, *Fusobacterium*, and *Akkermansia* were higher in the control group. Venn diagram showed that there were 121 common genera in both groups, 11 special genera, and 39 special genera in the control group and the CI group, respectively (Figure 3C). The results showed that there were significant differences in gut microbial composition between the two groups.

### Differential Taxa Between Groups

**Figure 4:**
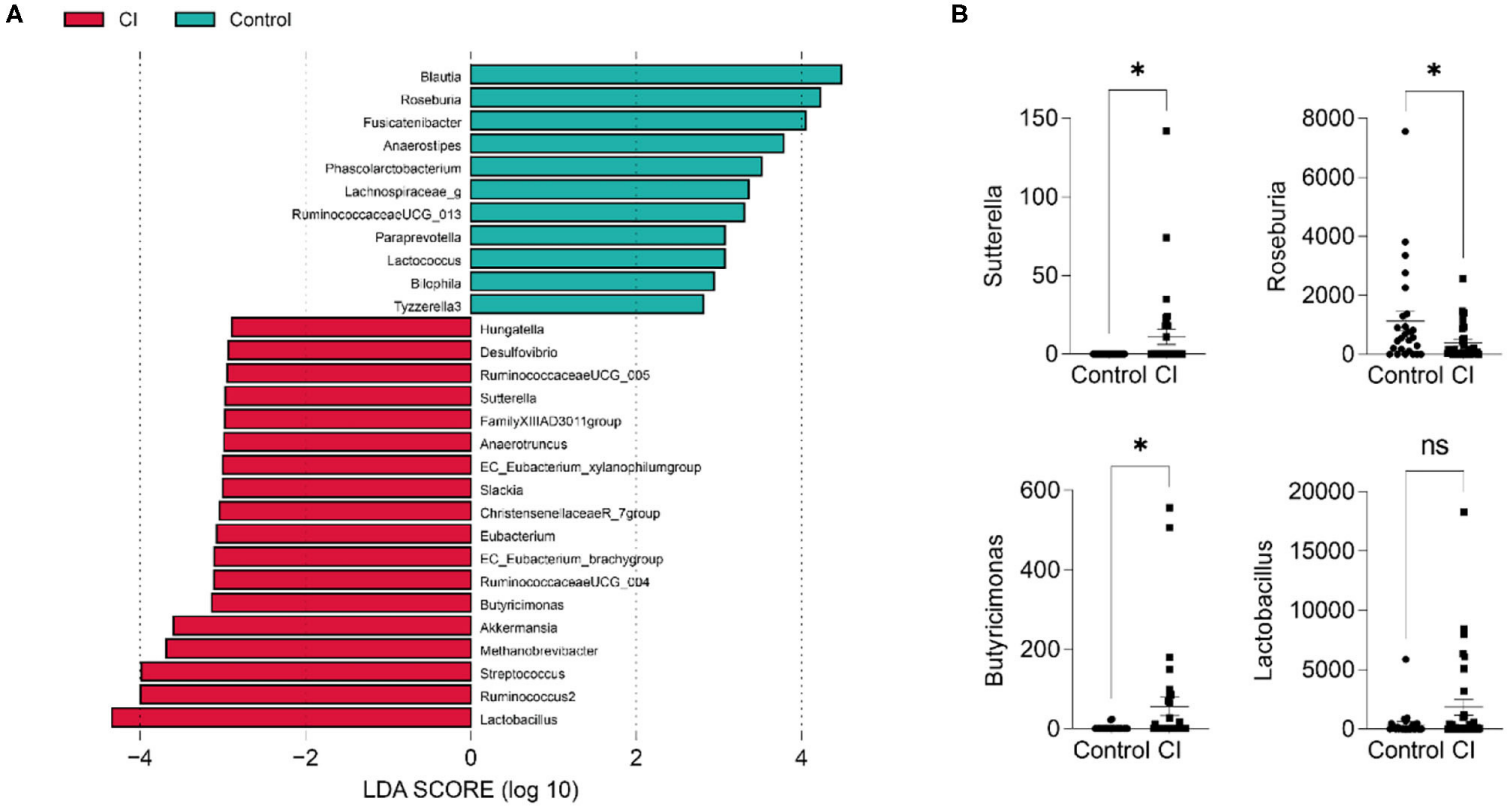
Differential taxa in the control and CI groups. **(A)** Linear discriminant analysis effect size (LEfSe). **(B)** Comparisons of 4 genera between groups (unpaired t-test, ns, no significance, ^*^p < 0.05).

To explore the differential taxa between the control and CI groups, LEfSe analysis was performed. The results showed that based on the alteration of relative abundances, 11 genera including *Blautia, Roseburia*, Fusicatenibacter, Anaerostipes, Phascolarctobacterium, Lachnospiraceae_g, RuminococcaceaeUCG_013, Paraprevotella, *Lactococcus*, Bilophila, and Tyzzerella 3 were enriched in the control group (Figure 4A). Hungatella, Desulfovibrio, RuminococcaceaeUCG_005, EC_Eubacterium_ xylanophilumgroup, FamilyXIIIAD3011group, Sutterella, Slackia, ChristensenellaceaeR_7group, Eubacterium, RuminococcaeaeUCG_004, Butyricimonas, *Akkermansia*, Methanobrevibacter, Streptococcus, *Ruminococcus*2, and *Lactobacillus* were the differential taxa in the CI group. For instance, *Sutterella* and *Butyricimonas* were higher in the CI group and Roseburia was higher in the control group. *Lactobacillus* was enriched in the CI group than that in the control group although there was no significant statistical significance (*p* > 0.05, Figure 4B).

### Gene Functions of Gut Microbiota Between Groups

**Figure 5:**
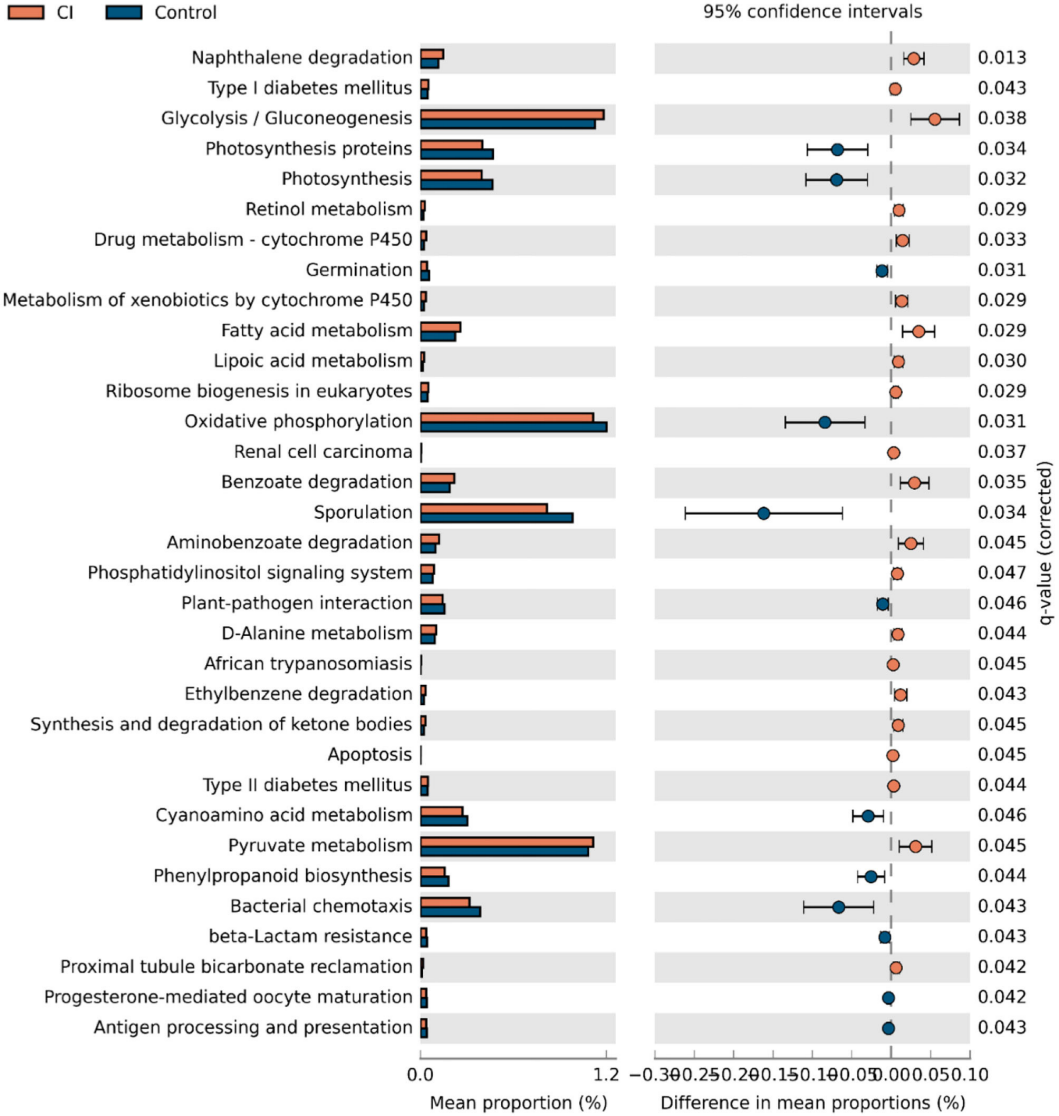
Alterations of gene functions of gut microbiota.

Gut microbial alterations lead to differences in gene functions. Therefore, to evaluate the changes in gene functions of gut microbiota, PICRUSt analysis was performed based on gene function prediction. There were 33 gene functions with significant alterations in both groups (Figure 5, two-sided, Welch's *t*-test with Benjamini-Hochberg FDR multiple test correction). The abundances of photosynthesis proteins, photosynthesis, germination, oxidative phosphorylation, sporulation, cyanoamino acid metabolism, phenylpropanoid biosynthesis, bacterial chemotaxis, beta-lactam resistance, progesterone-mediated oocyte maturation, and antigen processing and presentation were upregulated in the control group. However, gene functions related to nutrient metabolisms such as glycolysis/gluconeogenesis, retinol metabolism, fatty acid metabolism, D-alanine metabolism, and pyruvate metabolism were upregulated in patients with CI. The results showed that gut microbial alteration induced significant differences in gene functions and this might be one of the causes for CI.

### Identification of Microbial Markers for CI

**Figure 6:**
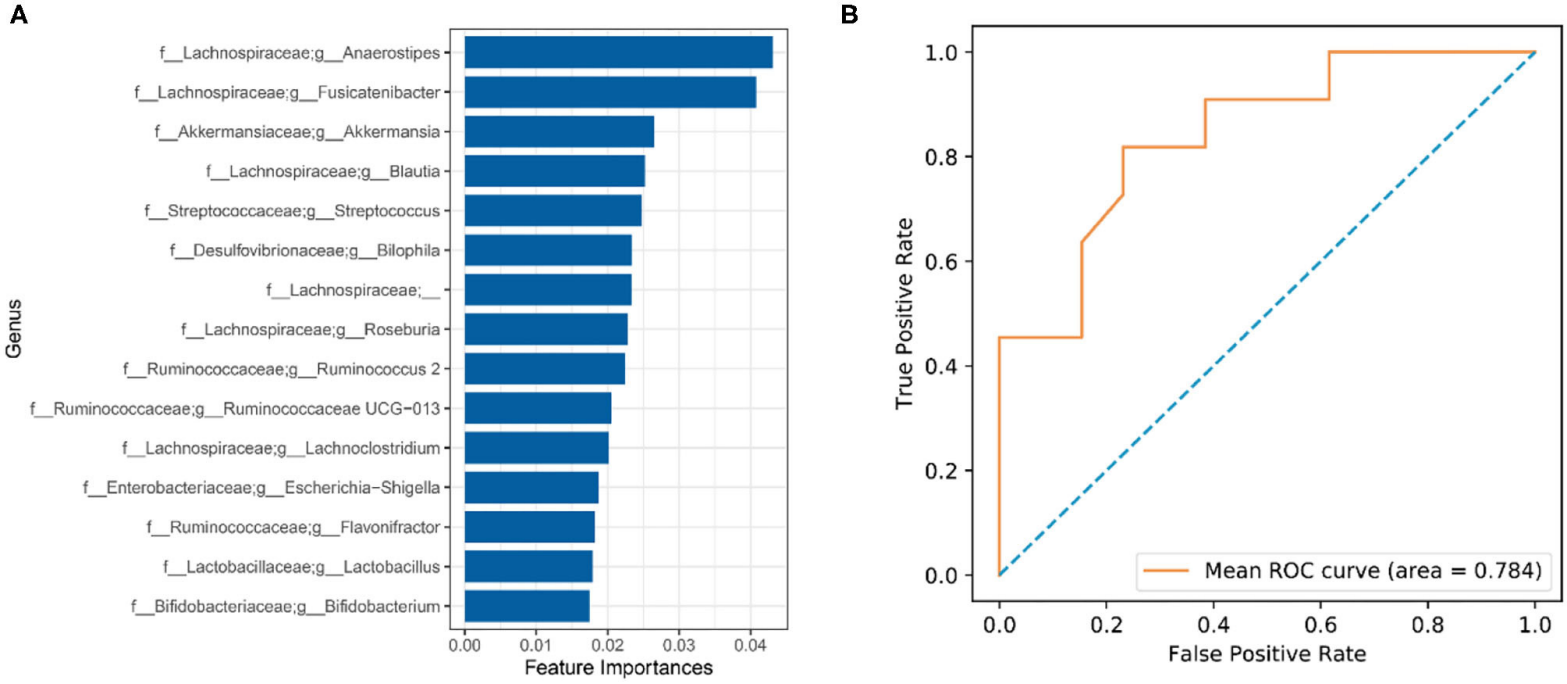
Identification of gut microbial markers for CI by random forest model. **(A)** Microbial markers were predicated using a random forest model. **(B)** ROC curve evaluation.

To explore gut microbial markers for CI, a random forest classifier model was used to identify the control samples from CI samples. To detect unique microbial markers of CI, a random forest model between 27 healthy samples and 33 CI samples in the discovery phase. As shown in Figure 6A, 15 microbial markers such as *Lactobacillus, Bifidobacterium*, and *Akkermansia* were selected as the optimal marker set. An area under curve (AUC) value of 78.4% was received in the ROC curve based on these selected genera and implied a high diagnosis efficacy for patients with CI (Figure 6B). The results showed that 15 genera contained more information for CI predication and might be the microbial markers for CI.

## Discussion

In the present study, gut microbiota from the patients with CI and control subjects was comprised using 16S rRNA sequencing. Gut microbial microecology is important for maintaining physiological functions in the host and contributes to the regulation of immune responses. Dysbiosis of gut microbiota leads to changes in metabolisms and results in the onset of diseases, especially including central nervous system diseases (22). Many studies have shown that modulating gut microbiota has been considered an effective target to alleviate emotional and behavioral disorders, such as depression, stroke, and autism (23–25). The increase in *Fusobacterium* and reduction of short-chain fatty acids have been revealed to be associated with post-stroke cognitive impairment (26), and this suggests the potential clinical diagnosis and treatment values of gut microbes for brain disorders.

Cognitive impairment (CI) is a common disease for the elderly, and the development of CI induces a decline in the ability of daily living to bring a huge burden to the family and society. Compared to the control group, patients with CI had a lower ADL and MMSE, although this might be partly attributed to the older age (Figure 1, *p* < 0.001). Furthermore, although in the CI group education duration was significantly higher than that in the control, that seemed to be no improvement for CI symptoms. Some studies have been shown education is negatively associated with CI performance, and more education duration time leads to an advantage of 0.2–0.4 *SD* in cognitive performance (27, 28). Increasing evidence has demonstrated that sleep disturbance is closely associated with the development of CI in the elderly. Long sleep latency leads to a decrease in the chance of reversion to normal cognition but long sleep duration is related to the decline of risk for cognition (29). However, patients with CI in this study had a more sleep duration time vs. the control subjects (Figure 1). This might be associated with the deficiency of assessment for sleep-related parameters such as midsleep time, sleep latency, and daytime dysfunction in this trial. Sleep duration did not mean good sleep quality for subjects. The sleep-wake cycle is important for brain aging and is a potential way to improve CI symptoms (30). Therefore, these results suggested that the older age, the higher risk for CI it was, and it was not related to education duration under ignoring education degree.

There were differences in gut microbiota between the control and CI groups. In the CI group, the Faith_pd index was significantly higher than that in the control but other characteristics related to alpha diversity had no significant differences (Figure 2A). Furthermore, gut microbial beta diversity had a significant difference between the two groups (*p* < 0.002, Figure 2B) and this suggested that patients with CI had a distinct structure and composition of gut microbes. Actinobacteria and Proteobacteria were increased but Bacteroidetes and Firmicutes were reduced in the CI group (Figure 3A). Patients with Alzheimer's disease had an increase in the abundance of Bacteroidetes but decreases in Firmicutes (31). Furthermore, *Bifidobacterium, Alistipes, Lactobacillus, Streptococcus*, [*Ruminococcus*] *torques* group, *Ruminococcus* 2, *Escherichia-Shigella*, and *Klebsiella* were increased in the CI group (Figure 3B). The proportions of *Bifidobacterium, Butyricicoccus*, and *Clostridium XIVb* were negatively correlated with the presence of CI in patients with Parkinson's disease (32). This was not consistent with our results. *Lactobacillus* and *Bifidobacterium* are common beneficial bacteria and have been demonstrated to involve in the synthesis of neurotransmitters such as acetylcholine, serotonin, and gamma-aminobutyric acid (32). *Escherichia-Shigella* is a conditional pathogen and is related to some diseases such as intestinal disorder and CI. An increase in the proportion of *Escherichia-Shigella* has been revealed to be associated with a peripheral inflammatory state in patients with CI and brain amyloidosis (33). Furthermore, patients with post-stroke CI had higher abundances of Enterococcus, Bacteroides, and *Escherichia-Shigella* and a lower Faecalibacterium vs. patients with stroke (34). Venn diagram showed there were special taxa in the control and CI groups and displayed the distinct gut microbial composition from each other (Figure 3C). Therefore, based on the differences in gut microbial composition, microbial markers might be explored for CI in the elderly.

Linear discriminant analysis effect size (LEfSe) revealed the differential taxa including *Desulfovibrio, Sutterella, Eubacterium, Butyricimonas, Akkermansia, Streptococcus*, and *Lactobacillus* in the CI group (Figure 4A). Compared to the control group, *Sutterella, Butyricimonas*, and *Lactobacillus* significantly increased in the CI group. APOE4 is a risk factor for Alzheimer's disease and APOE4 carrier has an increased rate of cognitive decline. A study has shown the abundance of *Sutterella* was significantly increased in male EFAD transgenic mice (35). Compared to the patients with Parkinson's disease having normal cognition, the proportions of *Blautia* and *Ruminococcus* were reduced (36). Our results also showed a low abundance of *Blautia* in the CI group. In a cohort trial study, *Parabacteroides, Verrucomicrobia, Akkermansia, Butyricimonas, Veillonella, Odoribacter, Mucispirillum, Bilophila, Enterococcus*, and *Lactobacillus* were significantly enriched in patients with Parkinson's disease than in controls (37), and some increased genera such as *Akkermansia, Butyricimonas*, and *Lactobacillus* were same to our findings.

We evaluated changes in gene functions of gut microbiota using PICRUSt analysis. The results showed that nutrient metabolisms such as glycolysis/gluconeogenesis, retinol metabolism, fatty acid metabolism, D-alanine metabolism, and pyruvate metabolism were upregulated in the CI group (Figure 5). The reduction of cognitive function is related to changes in brain glucose utilization (38). Compared to rats with normal cognition, significant differences in protein expression were demonstrated vs. the rats with CI and this was associated with the glycolysis/gluconeogenesis pathway. Supplement with ω-3 docosahexaenoic acid is an important way to prevent brain aging and the development of Alzheimer's disease (39), and thus increased fatty acid metabolism might be positively related to the development of CI. Furthermore, pyruvate has been reported to improve neuron survival and eliminate reactive oxygen species to alleviate CI symptoms in rats with Alzheimer's disease (40). Therefore, these studies were consistent with our findings, and upregulated metabolisms were positively related to CI symptoms. There were huge differences in gut microbial composition and gene functions, and thus gut microbiota had great potential to apply to microbial markers to diagnose the CI development. A random forest model was used to identify gut microbial markers for CI and the results showed that 15 genera were selected to predict the development of CI with an AUC value of 78.4% (Figure 6). Gut microbial markers have been applied to predict and diagnose early colorectal cancer and early hepatocellular carcinoma (15, 16). Prevention of CI is necessary for the elderly because it is difficult to reverse. Therefore, gut microbiota as a biomarker contributed to the diagnosis and prevention of CI in the elderly.

## Conclusion

Collectively, the abnormal gut microbial composition is associated with CI. A random forest model reveals the gut microbial markers of CI, and they have the potential to predict the development of CI and prevent CI based on gut microbial sequencing for the elderly.

## Data Availability Statement

The datasets presented in this study can be found in online repositories. The names of the repository/repositories and accession number(s) can be found in the article/ supplementary material.

## Ethics Statement

The studies involving human participants were reviewed and approved by the Ethics of Clinical Experiments of the Wuxi People's Hospital (KS2019039). The patients/participants provided their written informed consent to participate in this study.

## Author Contributions

SL performed experiments and wrote the manuscript. YZ and WL performed the study conceptualization. YY and QX performed data analysis and interpretation. ZW and BZ performed the data curation. JY contributed to the data acquisition and supervision. SW performed the data validation. KH developed the experimental design and revised the manuscript. All authors contributed to the article and approved the submitted version.

## Funding

This work was funded by the Young Project of the Wuxi Health Committee (Q201914), the Top Talent Support Program for Young and Middle-Aged People of Wuxi Health (BJ2020008), and the Major Project of the Wuxi Health Committee (Z202002).

## Conflict of Interest

The authors declare that the research was conducted in the absence of any commercial or financial relationships that could be construed as a potential conflict of interest.

## Publisher's Note

All claims expressed in this article are solely those of the authors and do not necessarily represent those of their affiliated organizations, or those of the publisher, the editors and the reviewers. Any product that may be evaluated in this article, or claim that may be made by its manufacturer, is not guaranteed or endorsed by the publisher.
